# Supplementary material for: Vibrio cholerae biofilms use modular adhesins with glycan-targeting and nonspecific surface binding domains for colonization
Source: Nat Commun. 2023 Apr 13;14:2104. doi: 10.1038/s41467-023-37660-0 (PMC10102183; doi:10.1038/s41467-023-37660-0)
Supplement: Supplementary file 1 — Supplementary Information [file 41467_2023_37660_MOESM1_ESM.pdf]

Supplementary Information for

Vibrio cholerae biofilms use modular adhesins with glycan-targeting and nonspecific surface binding domains for colonization

Xin Huang,[1,2,#] Thomas Nero,[1,#] Ranjuna Weerasekera,[3] Katherine Matej,[1] Alex Hinbest,[3] Zhaowei Jiang,[1] Rebecca F. Lee,[4] Longjun Wu,[5] Cecilia Chak,[1] Japinder Nijjer,[1] Isabella Gibaldi,[3] Hang Yang,[3] Nathan Gamble,[3] Wai-Leung Ng,[6] Stacy A. Malaker,[2] Kaelyn Sumigray[4,7,8], Rich Olson,[3,*] Jing Yan[1,9,*]

[1]Department of Molecular, Cellular and Developmental Biology, Yale University, New Haven, CT, USA.

[2]Department of Chemistry, Yale University, New Haven, CT, USA.

[3]Department of Molecular Biology and Biochemistry, Molecular Biophysics Program, Wesleyan University, Middletown, CT, USA.

[4]Department of Genetics, Yale School of Medicine, New Haven, CT, USA.

[5]Department of Ecology and Evolutionary Biology, Yale University, New Haven, CT, USA.

[6]Department of Molecular Biology and Microbiology, Tufts University School of Medicine, Boston, MA, USA.

[7]Yale Stem Cell Center, Yale School of Medicine, New Haven, CT, USA.

[8]Yale Cancer Center, Yale School of Medicine, New Haven, CT, USA.

[9]Quantitative Biology Institute, Yale University, New Haven, CT, USA.

[#]These authors made equal contributions

*Correspondence: rolson@wesleyan.edu (R.O.), jing.yan@yale.edu (J.Y.)

This PDF includes:

Supplementary Figures 1-9; Supplementary Tables 1-2

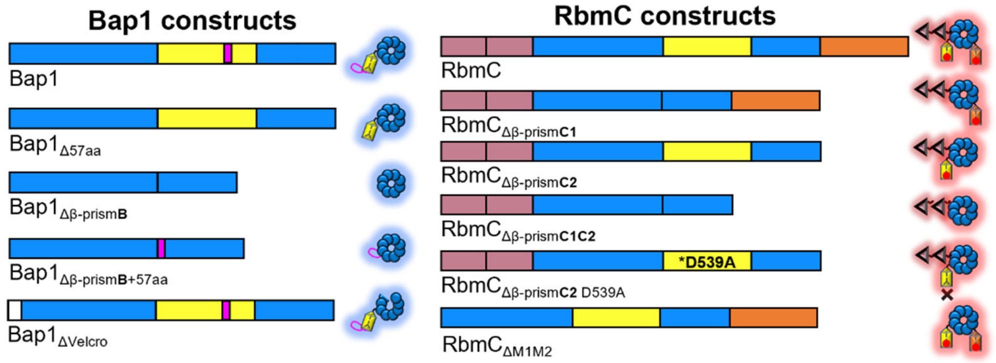

Supplementary Figure 1| Schematic of domains and corresponding cartoon representations of Bap1 (*Left*) and RbmC (*Right*) mutants used in this study.

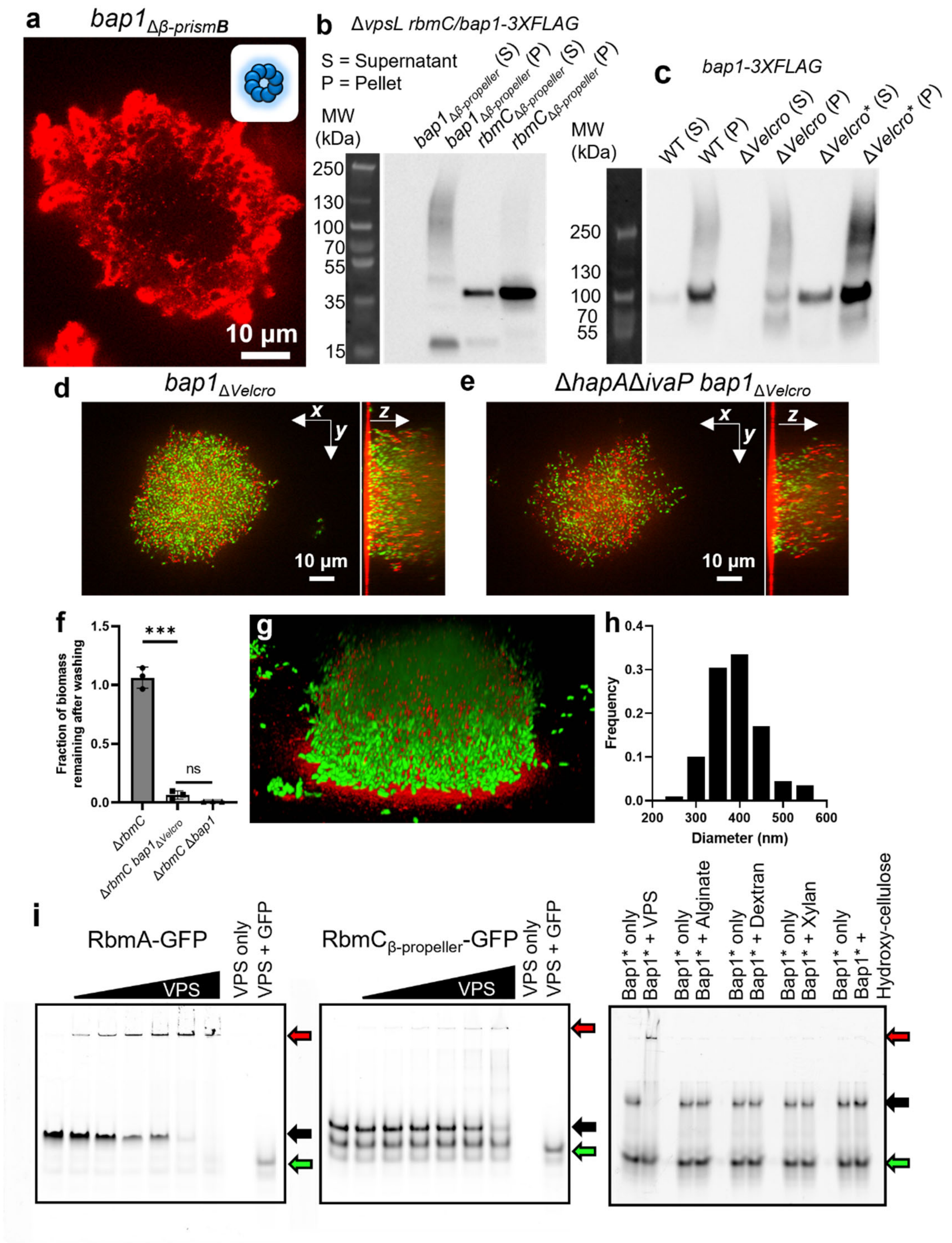

Supplementary Figure 2| β-propeller anchors Bap1 and RbmC to VPS. a, Cross-sectional images of $bap1_{\Delta\beta\text{-}prismB}$ at z = 6 µm. Cells constitutively express mNeonGreen; Bap1 was stained with an anti-FLAG antibody conjugated to Cy3. $Bap1_{\Delta\beta\text{-}prismB}$ retains peripheral staining around biofilm clusters, suggesting that VPS binding only requires the β-propeller. **b**, Western blot analysis of the production and secretion of constructs missing the β-propeller in the VPS^{-} (Δ*vpsL*) background. A significant fraction of the proteins is found in the cell pellet, indicating a defect in

secretion. Therefore, these mutants were excluded from further analysis. **c**, Western blot of *V. cholerae* strains expressing 3×FLAG-tagged WT or $Bap1_{\Delta Velcro}$ protein (* denotes strain lacking *hapA* and *ivaP*). In the WT background, $Bap1_{\Delta Velcro}$ experiences degradation leading to lower signal intensity. Deleting *hapA* and *ivaP*, the two major extracellular proteases[1], resolves the degradation problem. **d-e,** Cross-sectional images of $bap1_{\Delta Velcro}$ (d) and Δ*hapA*Δ*ivaP* $bap1_{\Delta Velcro}$ (e) in the biofilm bulk (z = 6 μm). Cells constitutively express mNeonGreen; Bap1 was stained with an anti-FLAG antibody conjugated to Cy3. Note that a functional copy of RbmC is present in these strains. Deletion of *hapA* and *ivaP* does not change the localization or aggregation of the mutant protein. **f,** Biofilm adhesion assay with $bap1_{\Delta Velcro}$, together with positive (Δ*rbmC*) and negative (Δ*rbmC*Δ*bap1*) controls. Data are shown as the mean ± SD (n = 3 biologically independent samples). Statistics were performed using an unpaired, two-tailed *t*-test with a Welch's correction. ns represents not significant (Δ*rbmC* $bap1_{\Delta Velcro}$ v.s. Δ*rbmC*Δ*bap1*: p = 0.1144) ; *** $p < 0.001$ (Δ*rbmC* v.s. Δ*rbmC* $bap1_{\Delta Velcro}$: $p = 0.0009$). **g**, 3D-rendering of confocal images of a biofilm from $Bap1_{\Delta velcro}$-3×FLAG cells expressing mNeonGreen with *in situ* immunostaining. **h**, Distribution of puncta size in $bap1_{\Delta Velcro}$ biofilms (N = 200 technical replicates). **i**, EMSA of RbmA (*Left*) binding to VPS, RbmC's β-propeller binding to VPS (*Middle*), and Bap1*(= $Bap1_{\Delta 57aa}$, *Right*) binding to selected polysaccharides. RbmA was used as a positive control in the EMSA as it is known to bind to VPS[2]. The protein amount per lane is 5 μg when present. The VPS amount is 0, 0.0625, 0.125, 0.25, 0.50, 1, 5, 5, 5 μg in each lane from left to right. For other polysaccharides, 5 μg is used in each lane whenever present. Red arrows = protein-substrate complex, black arrows = unbound protein, green arrows = free GFP. Under the concentrations tested, we did not observe the same tight binding of Bap1 to the selected polysaccharides as seen with VPS.

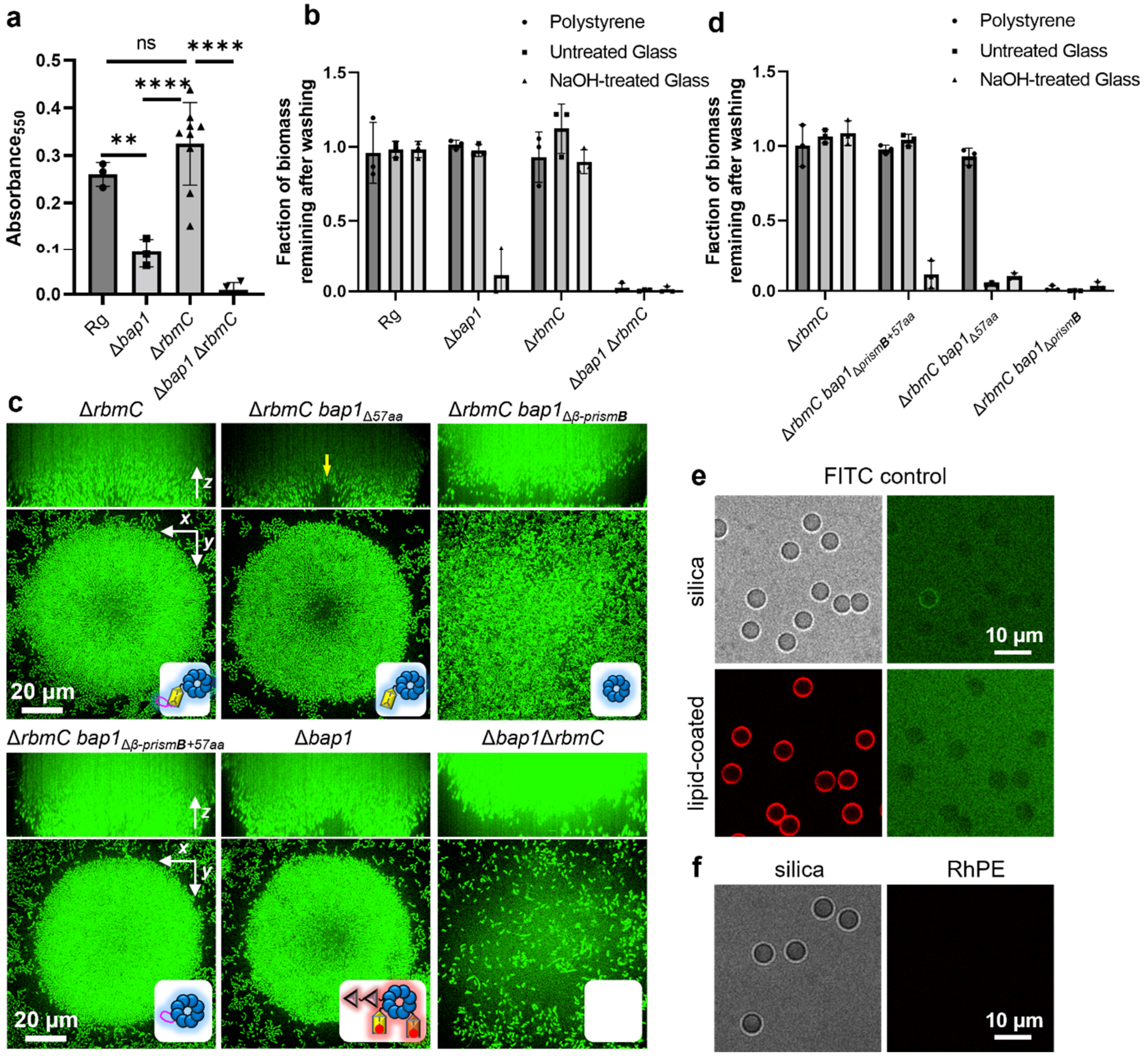

Supplementary Figure 3| Bap1 is the major biofilm adhesin on abiotic surfaces. a, Absorbance at 550 nm in crystal violet assays for the indicated strains. Data are shown as the mean ± SD (n = 3 for rugose and Δ*rbmC*, n = 4 for Δ*bap1*Δ*rbmC*, n = 9 for Δ*rbmC*, all biologically independent samples). Statistics were performed using an unpaired, two-tailed t-test with a Welch's correction. ns represents not significant (Rg v.s. Δ*rbmC*: $p = 0.0751$); ** $p < 0.01$ (Rg v.s. Δ*bap1*: $p = 0.0026$); **** $p < 0.0001$. **b**, Biofilm adhesion assays performed on polystyrene, untreated glass, or NaOH-treated glass (following an increasing order of hydrophilicity), for various mutants with a single or double deletion of *bap1* and/or *rbmC*. Data are shown as the mean ± SD (n = 3 biologically independent samples). On NaOH-treated glass that is more hydrophilic and negatively charged[3], a single deletion of *bap1*, but not *rbmC*, is sufficient to lead to an adhesion defect. **c,** Representative cross-sectional views of the bottom layer and side views of different mutant biofilms. The two defective mutants Δ*bap1*Δ*rbmC* and Δ*rbmC bap1*$_{\Delta\beta\text{-}prism\mathbf{B}}$ form floating clusters not attached to the surface. The Δ*rbmC bap1*$_{\Delta 57aa}$ displays a hole at biofilm core (yellow arrow), indicating a slight adhesion defect. **d**, Biofilm adhesion assays performed on polystyrene, untreated glass, or NaOH-treated glass, for various *bap1* mutants in the presence of 1 mg/mL BSA. Data are shown as the

mean ± SD (n = 3 biologically independent samples). The Bap1$_{\Delta\beta\text{-prism}\mathbf{B}+57aa}$ construct, although functional on bare glass, does not adhere to NaOH-treated glass, suggesting that β-prism**B** still contributes to abiotic surface adhesion to some extent. **e**, Negative control for the microbead adsorption assay. *Top*: a representative image of 5 μm silica bead (*Left*, bright field) and FITC adsorbed on the bead (*Right*). *Bottom*: a representative image of 5 μm silica beads coated with lipids, labeled with RhPE (*Left*) for lipids and the FITC adsorbed on lipid layer (*Right*). [FITC] = 1.5 μM. FITC molecules adsorb minimally to these two types of surfaces. See main Figure 3f for quantification. **f**, A representative image of 5 μm silica beads incubated with RhPE. RhPE does not adsorb onto silica surfaces unless coated with lipids. The intensity scale in e and f is the same as that in main Figure 3e.

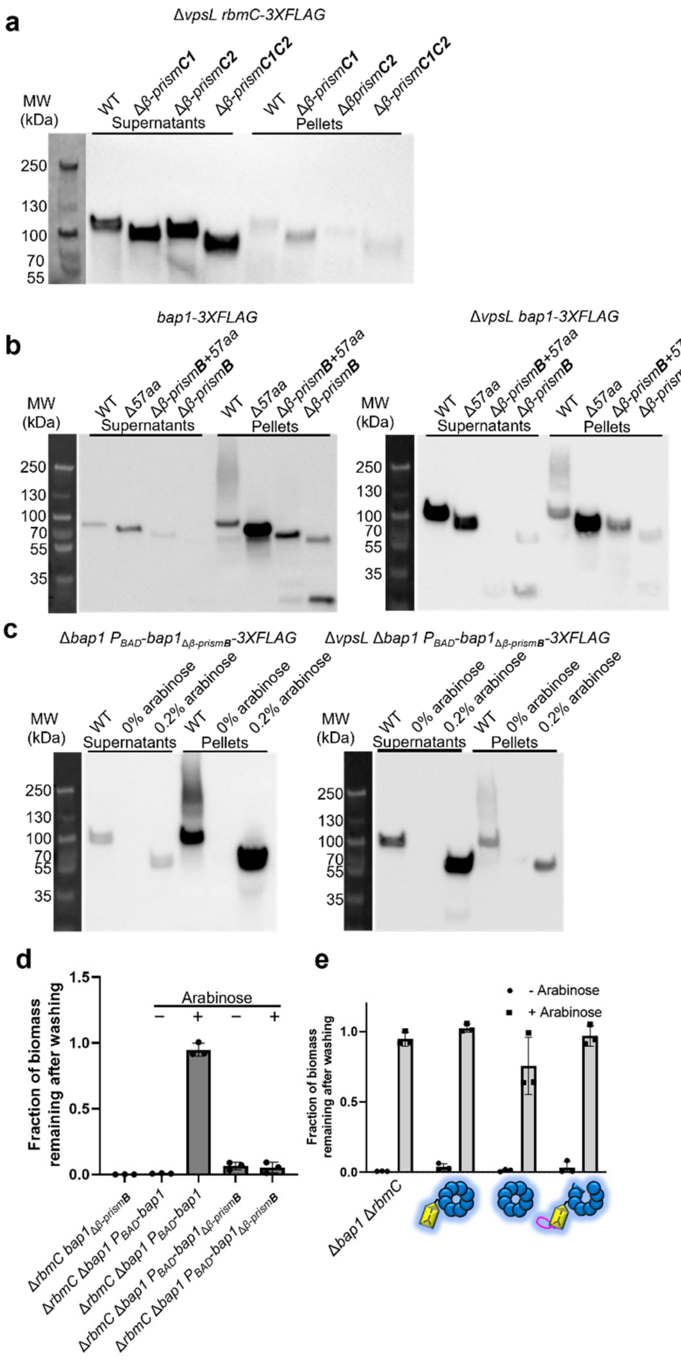

Supplementary Figure 4| Analysis of protein stability and secretion of Bap1 and RbmC constructs. **a**, Western blot analysis of the production and secretion of RbmC protein constructs in VPS^{-} *V. cholerae* strains. All constructs are secreted into the supernatant at a level comparable to WT. **b**, Western blot analysis of the production and secretion of Bap1 protein constructs in VPS^{+} (*Left*) and VPS^{-} (*Right*) *V. cholerae* strains. Among the defective mutants, Bap1$_{\Delta\beta\text{-prism}\mathbf{B}}$ shows reduced intensity due to degradation and potentially also secretion. To verify that the defective phenotype seen with Bap1$_{\Delta\beta\text{-prism}\mathbf{B}}$ is not due to these confounding factors, we overexpressed *bap1*$_{\Delta\beta\text{-}prism\mathbf{B}}$ from an arabinose-inducible plasmid. **c**, Western blot analysis of induction,

production, and secretion of *bap1*$_{\Delta\beta\text{-}prismB}$-3×FLAG from a plasmid in the VPS^{+} (*Left*) and VPS^{-} (*Right*) background, with and without 0.2% arabinose. In the presence of arabinose, the level of secreted proteins is restored to a level higher to that of WT Bap1. **d**, Adhesion assay of the strain overexpressing *bap1*$_{\Delta\beta\text{-}prismB}$ in the presence of 1 mg/mL BSA. Data are shown as the mean ± SD (*n* = 3 biologically independent samples). Even in the presence of arabinose that leads to elevated levels of the secreted protein, *bap1*$_{\Delta\beta\text{-}prismB}$ is unable to adhere the biofilm to the glass surface, suggesting that the protein is inherently defective. **e,** Adhesion defective mutants can be complemented. Shown are results from adhesion assay (from left to right) of Δ*rbmC*Δ*bap1*, Δ*rbmC bap1*$_{\Delta Velcro}$, Δ*rbmC bap1*$_{\Delta 57aa}$, *and* Δ*rbmC bap1*$_{\Delta\beta\text{-}prismB}$ mutants with a plasmid containing P_{BAD}-*bap1* induced with 0.2% arabinose in the presence of 1 mg/mL BSA. Data are shown as the mean ± SD (*n* = 3 biologically independent samples). The complementation assay validates that the defective phenotype of these strains is due to the specific mutations in Bap1.

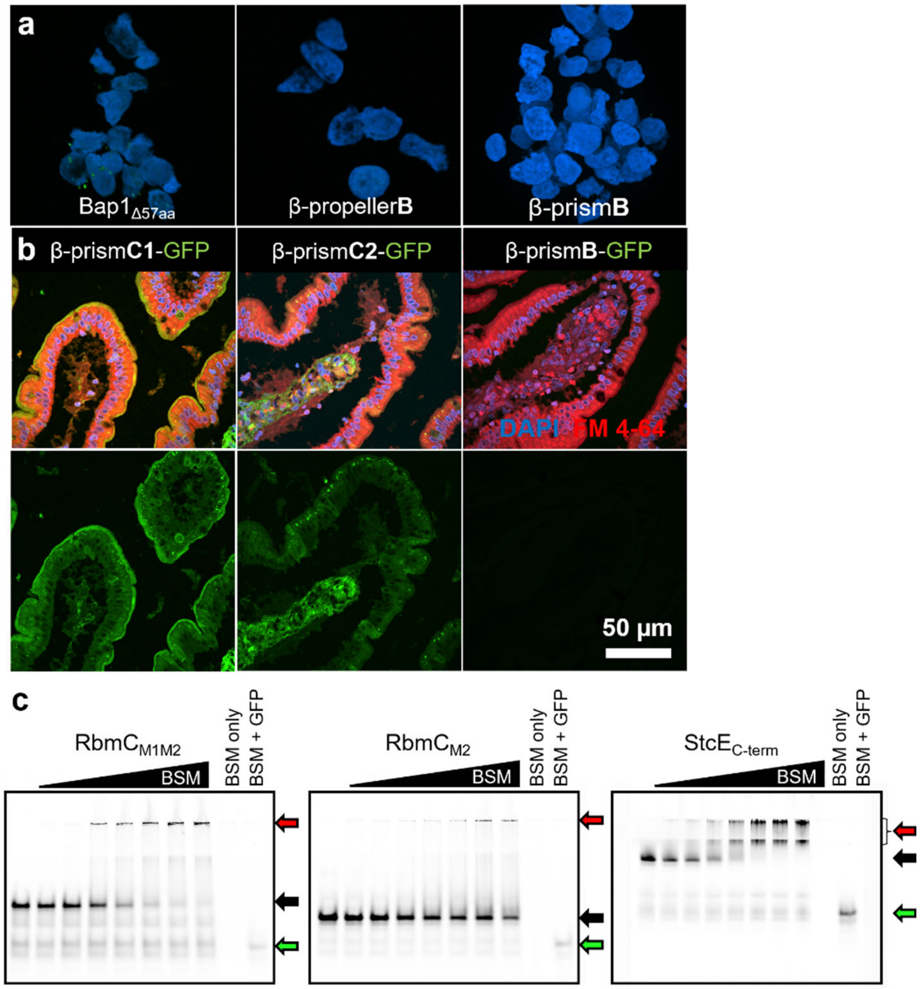

Supplementary Figure 5| Purified functional RbmC constructs but not Bap1 constructs bind to glycans prevalent on host surfaces. a, Merged z-stack of confocal images of DAPI-stained Caco-2 cells incubated with 1 μM purified and GFP-tagged Bap1 domains. The total size of each image is 80×80×32.5 μm. The intensity scale is the same as that in Figure 4a. None of the Bap1 domain(s) shows positive staining with Caco-2 cells. **b**, Confocal images of jejunum tissue slices stained with DAPI, FM 4-64, and 1 μM purified and GFP-tagged β-prism**C1** (*Left*), β-prism**C2** (*Middle*), and β-prism**B** (*Right*). **c**, EMSA of $RbmC_{M1M2}$ (*Left*), $RbmC_{M2}$ (*Middle*), or $StcE_{C\text{-}term}$ (*Right*) binding to bovine submaxillary mucin (BSM). The protein amount per lane is 5 μg when present. The BSM amount is 0, 0.5, 1, 2, 4, 6, 8, 10, 10, 10 μg in each lane from left to right. Red arrows = protein-substrate complex, black arrows = unbound protein, green arrows = free GFP.

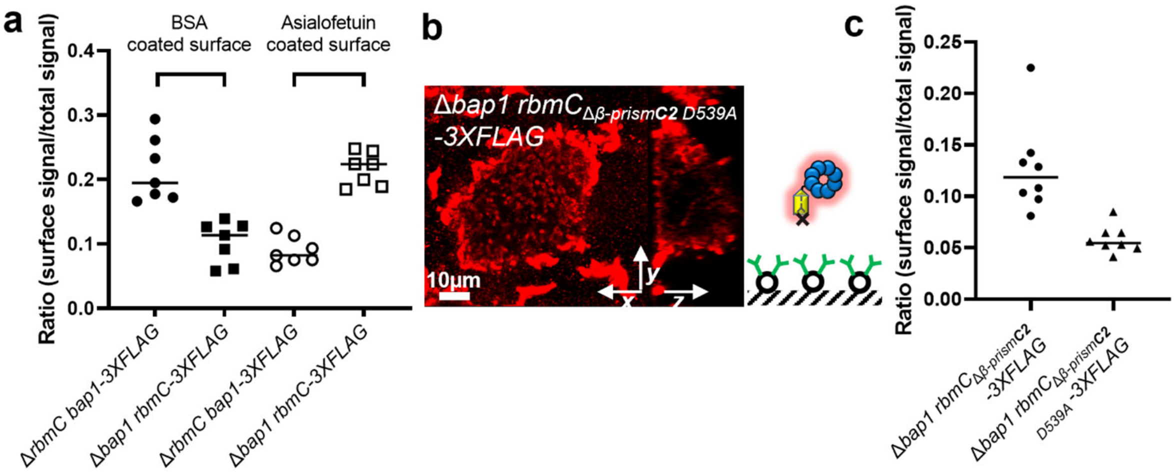

Supplementary Figure 6| Role of β-prism domains in adhering *V. cholerae* biofilms and directing adhesins to bare and functionalized glass surfaces. a, Distribution quantification for Bap1 and RbmC in the presence of BSA or asialofetuin. Shown are the ratios between the signals of 3×FLAG-tagged proteins at the biofilm-glass interface and the total signal integrated over the entire biofilm cluster, for each indicated strain. The relative order of the two proteins localized to the biofilm-glass interface is opposite in the two cases, showing different preferences of the two adhesins for different types of surfaces. **b-c,** Asialofetuin binding requires a functional β-prism. **b**, Cross-sectional image of a biofilm from Δ*bap1 rbmC*$_{Δβ\text{-}prism\mathbf{C2}\ D539A}$, in which N-glycan binding was abolished due to a point mutation in a key aspartate residue in the binding pocket[4] (schematically shown on the right). The surface localization of RbmC signal is lost due to the loss of N-glycan binding, as quantified in panel **c**. For **a** and **c**, individual points are from different biofilms in one sample (technical replicates), and no statistics were derived.

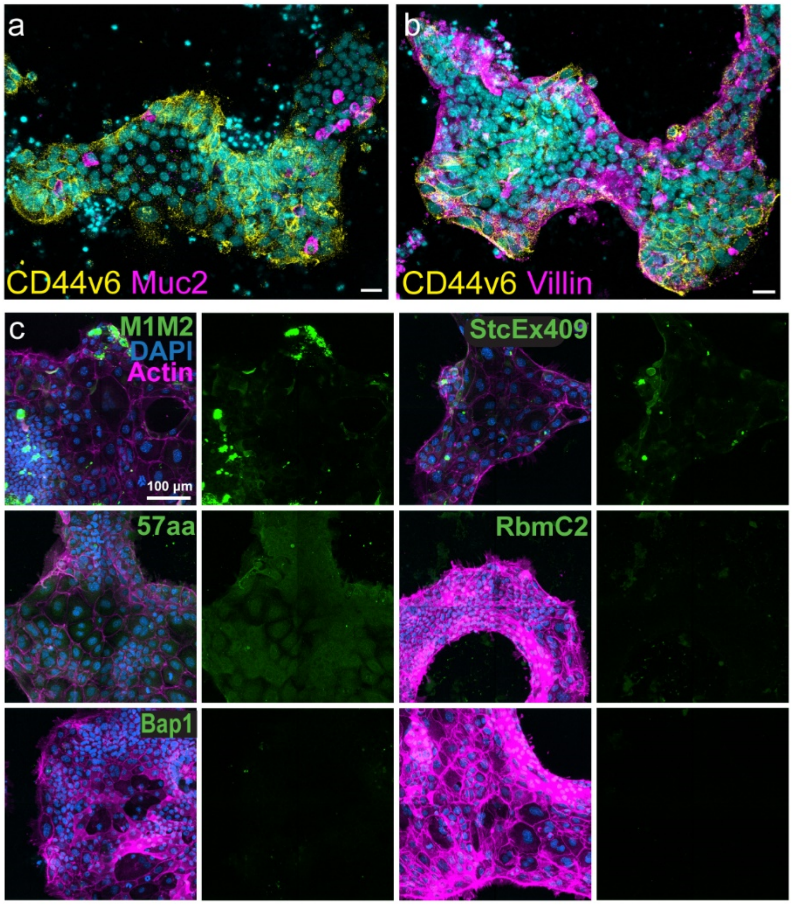

Supplementary Figure 7| Validation of enteroid monolayers. Shown are maximum projection images of a representative monolayer with nuclei stained with DAPI (cyan), crypt domains stained with CD44v6 antibody (yellow), goblet cells stained with MUC2 antibody (magenta) in **a** and enterocytes stained with Villin (magenta) in **b**. Scale bars: 20 μm. **c,** Representative maximum projection images of enteroid monolayers stained with nuclei stained with DAPI (blue), an F-actin probe conjugated to Alexa Fluor^{TM} 647 dye (magenta), and 1 μM labeled proteins. All proteins are labeled with GFP_{UV}, and the 57aa peptide is labeled with FITC. The bottom right panel shows a control without any protein/peptide staining. Shown on the left column are the overlay images of all three fluorescent channels and on the right column are the signals in the 488 nm channel.

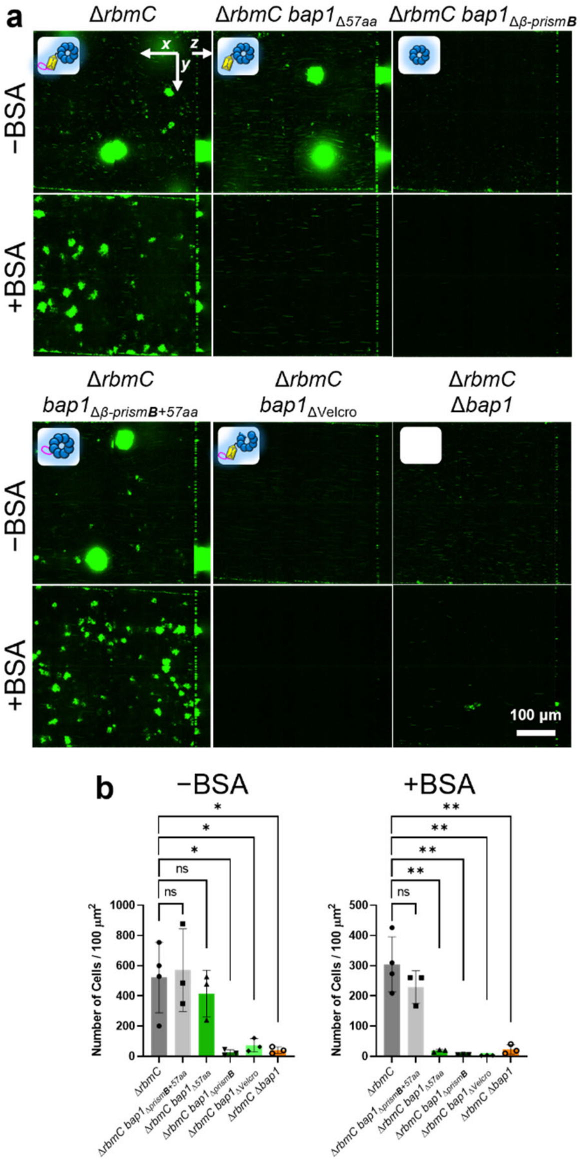

Supplementary Figure 8| Validation of the role of the biofilm adhesins in the wild-type background. a, WT *V. cholerae* biofilms with different constructs grown in microfluidic chambers under a flow rate of 0.6 µL/min. Shown are both cross-sectional views of biofilms at z = 6 µm, both in the absence (*Top*) and presence (*Bottom*) of 0.4 mg/mL BSA. **b,** Quantification of biomass for different WT biofilms (mean ± SD, n (biologically independent samples) = 4 for Δ*rbmC* and n = 3 for all other mutants, two-tailed t-test with Welch's correction. *$p < 0.05$, **$p < 0.01$, ns = not significant). p values from left to right (in the absence of BSA): 0.8139, 0.5010, 0.0232, 0.0284, 0.0253. p values from left to right (in the presence of BSA): 0.2340, 0.0081, 0.0072, 0.0071, 0.0072. Note that the surface layer was excluded from the analysis; WT *V. cholerae* cells can attach to glass surfaces via pili and form a monolayer independent of their ability to form biofilms[5].

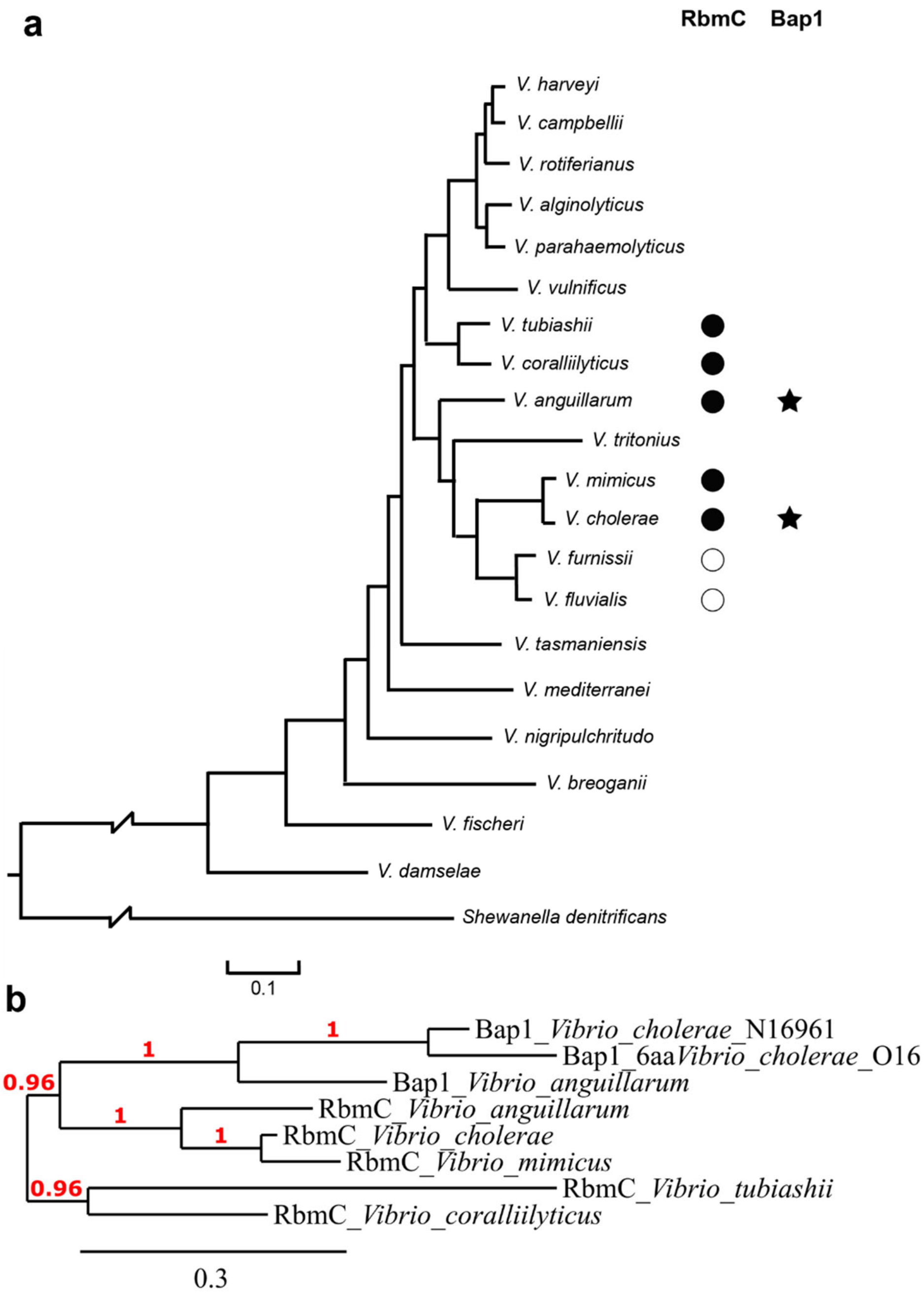

Supplementary Figure 9| Phylogenetic analysis of RbmC and Bap1 among Vibrio species. a, Phylogenetic tree of 20 Vibrio species adapted from Ref.6, showing the distribution of RbmC/Bap1 homologues. The branch lengths are approximate. Filled circles indicate where a RbmC gene was recovered. Open circles indicate where a potential RbmC gene with low conservation was recovered. Stars indicate where Bap1 was recovered. Scale bar: the number of substitutions per site. **b**, Phylogenetic analysis of Bap1 and RbmC homologs in Vibrio species. Scale bar: number of amino acid substitutions per site.

Supplementary Table 1- Strains used in this study

Strain Name in Manuscript	Genotype and Antibiotic Resistance	Description	Strain# & Reference
WT background	C6706, Sm^R	A streptomycin-resistant variant of the WT O1 El Tor biotype C6706str2. Serves as the background for all strains reported in this manuscript	JY016[7]
Rg background	*vpvC*^{W240R}, Sm^R	Missense mutation in the *Vibrio cholerae* O1 El tor strain that elevates the level of cyclic-di-GMP. Rugose phenotype. Serves as the parental strain for most of the mutants in this manuscript	JY028[8]
**Strains Used for Biofilm Morphology and Adhesion Assays**			
Rg	*vpvC*^{W240R}, *ΔVC1807*::*P*$_{tac}$-*mNeonGreen*, $Spec^R$	Rugose strain with mNeonGreen fluorescent protein inserted at a non-essential site in the genome (VC1807)	JY451[9]
Δbap1	*vpvC*^{W240R}, *Δbap1*, *ΔVC1807*::*P*$_{tac}$-*mNeonGreen*, $Spec^R$	Clean deletion of *bap1* by cotransformation with *ΔVC1807*::*P*$_{tac}$-*mNeonGreen*, $Spec^R$	ZJ053, This Study
ΔrbmC	*vpvC*^{W240R}, *ΔrbmC*, *ΔVC1807*::*P*$_{tac}$-*mNeonGreen*, $Spec^R$	Clean deletion of *rbmC* by cotransformation with *ΔVC1807*::*P*$_{tac}$-*mNeonGreen*, $Spec^R$	STN0011, This Study
ΔvpsL	*vpvC*^{W240R}, *ΔvpsL*, *ΔVC1807*::*P*$_{tac}$-*mNeonGreen*, $Spec^R$	Clean deletion of *vpsL* by cotransformation with *ΔVC1807*::*P*$_{tac}$-*mNeonGreen*, $Spec^R$	JY466, This Study
Δbap1 ΔrbmC	*vpvC*^{W240R}, *Δbap1*, *ΔrbmC*, *ΔVC1807*::*P*$_{tac}$-*mNeonGreen*, $Spec^R$	Clean deletion of *rbmC* by cotransformation with *ΔVC1807*::*P*$_{tac}$-*mNeonGreen*, $Spec^R$ into a rugose strain lacking *bap1* (JY074)	STN0009[10]
ΔrbmC bap1$_{Δβ\text{-}prism\mathbf{B}}$	*vpvC*^{W240R}, *ΔrbmC*, *bap1*$_{Δβ\text{-}prism\mathbf{B}}$, *ΔVC1807*::*P*$_{tac}$-*mNeonGreen*, $Spec^R$	Replacement of *bap1*^{WT} with a *bap1* construct lacking the β-prism domain by cotransformation with *ΔVC1807*::*P*$_{tac}$-*mNeonGreen*, $Spec^R$ into a rugose strain lacking *rbmC* (JY071)	ZJ074, This Study

Δ*rbmC bap1*$_{\Delta 57aa}$	*vpvC*^{W240R}, Δ*rbmC, bap1*$_{\Delta 57aa}$, Δ*VC1807*::P_{tac}-*mNeonGreen,* Spec^R	Replacement of *bap1*^{WT} with a *bap1* construct lacking the 57aa by cotransformation with Δ*VC1807*::P_{tac}-*mNeonGreen,* Spec^R into a rugose strain lacking *rbmC* (JY071)	ZJ032, This Study
Δ*rbmC bap1*$_{\Delta\beta\text{-}prism\mathbf{B}+57aa}$	*vpvC*^{W240R}, Δ*rbmC, bap1*$_{\Delta\beta\text{-}prism\mathbf{B}+57aa}$, Δ*VC1807*::$P_{tac}$-*mNeonGreen*, Spec^R	Replacement of *bap1*^{WT} with a *bap1* construct lacking the prism but with remaining 57aa by cotransformation with Δ*VC1807*::P_{tac}-*mNeonGreen,* Spec^R into a rugose strain lacking *rbmC* (JY071)	ZJ087, This Study
Δ*rbmC bap1*$_{\Delta Velcro}$	*vpvC*^{W240R}, Δ*rbmC, bap1*$_{\Delta Velcro}$, Δ*VC1807*::P_{tac}-*mNeonGreen*, Spec^R	Replacement of *bap1*^{WT} with a *bap1* construct lacking the Velcro by cotransformation with Δ*VC1807*::P_{tac}-*mNeonGreen,* Spec^R into a rugose strain lacking *rbmC* (JY071)	XH031, This Study
Δ*rbmC bap1*$_{\Delta\beta\text{-}propeller\mathbf{B}}$	*vpvC*^{W240R}, Δ*rbmC, bap1*$_{\Delta\beta\text{-}propeller\mathbf{B}}$, Δ*VC1807*::$P_{tac}$-*mNeonGreen*, Spec^R	Replacement of *bap1*^{WT} with a *bap1* construct lacking the β-propeller domain by cotransformation with Δ*VC1807*::P_{tac}-*mNeonGreen,* Spec^R into a rugose strain lacking *rbmC* (JY071)	XH001, This Study
Δ*rbmC* Δ*bap1* +P_{BAD}-*bap1*$_{\Delta\beta\text{-}prism\mathbf{B}}$	*pEVS*(P_{BAD}_*bap1*$_{\Delta\beta\text{-}prism\mathbf{B}}$, Kan^R), *vpvC*^{W240R}, Δ*rbmC,* Δ*bap1,* Δ*VC1807*::P_{tac}-*mNeonGreen,* Spec^R	*pEVS* containing *bap1*$_{\Delta\beta\text{-}prism\mathbf{B}}$ with an arabinose inducible promoter was mated into the indicated strain for overexpression	XH088, This Study
Δ*bap1 rbmC*$_{\Delta\beta\text{-}prism\mathbf{C1}}$	*vpvC*^{W240R}, Δ*bap1, rbmC*$_{\Delta\beta\text{-}prism\mathbf{C1}}$, Δ*VC1807*::$P_{tac}$-*mNeonGreen,* Spec^R	Replacement of *rbmC*^{WT} with a *rbmC* construct lacking the first β-prism domain by cotransformation with Δ*VC1807*::P_{tac}-*mNeonGreen,* Spec^R into a rugose strain lacking *bap1* (JY074)	STN0014, This Study
Δ*bap1 rbmC*$_{\Delta\beta\text{-}prism\mathbf{C2}}$	*vpvC*^{W240R}, Δ*bap1, rbmC*$_{\Delta\beta\text{-}prism\mathbf{C2}}$, Δ*VC1807*::$P_{tac}$-*mNeonGreen*, Spec^R	Replacement of *rbmC*^{WT} with a *rbmC* construct lacking the second β-prism domain by cotransformation with Δ*VC1807*::P_{tac}-*mNeonGreen,* Spec^R into a rugose strain lacking *bap1* (JY074)	STN0015, This Study
Δ*bap1 rbmC*$_{\Delta\beta\text{-}prism\mathbf{C1C2}}$	*vpvC*^{W240R}, Δ*bap1, rbmC*$_{\Delta\beta\text{-}prism\mathbf{C1C2}}$, Δ*VC1807*::$P_{tac}$-*mNeonGreen,* Spec^R	Replacement of *rbmC*^{WT} with a *rbmC* construct lacking both β-prism domains by cotransformation with Δ*VC1807*::P_{tac}-*mNeonGreen,* Spec^R into a rugose strain lacking *bap1* (JY074)	STN0024, This Study

Δ*bap1 rbmC* ΔM1M2	*vpvC*^{W240R}*, Δbap1, rbmC*$_{ΔM1M2}$, Δ*VC1807*::*P*$_{tac}$-*mNeonGreen,* Kan^R	Replacement of *rbmC*^{WT} with a *rbmC* construct lacking the M1M2 domain by cotransformation with Δ*VC1807*::*P*$_{tac}$*-mNeonGreen,* Spec^R into a rugose strain (JY028). Strain was conjugated with a plasmid containing a clean *bap1* deletion (pJY53). Due to weak fluorescence, strain was transformed with Δ*VC1807*::*P*$_{tac}$*-mNeonGreen,* Kan^R	SKH0058, This Study
WT Δ*rbmC*	Δ*rbmC,* Δ*VC1807*::*P*$_{tac}$-*mNeonGreen*, Spec^R	Transformation with Δ*VC1807*::*P*$_{tac}$-*mNeonGreen,* Spec^R into a WT strain lacking *rbmC* (JY063)	XH120, This Study
WT Δ*rbmC* Δ*bap1*	Δ*rbmC,* Δ*bap1,* Δ*VC1807*::*P*$_{tac}$-*mNeonGreen*, Spec^R	Transformation with Δ*VC1807*::*P*$_{tac}$-*mNeonGreen,* Spec^R into a WT strain lacking *bap1* and *rbmC* (JY067)	XH121, This Study
WT Δ*rbmC* *bap1*$_{Δ57aa}$	Δ*rbmC, bap1*$_{Δ57aa}$*,* Δ*VC1807*::*P*$_{tac}$-*mNeonGreen*, Kan^R	Converting rugose strain to WT background by cotransformation of WT *vpvc* locus with Δ*VC1807*::*P*$_{tac}$-*mNeonGreen,* Kan^R into a rugose strain lacking *57aa* (ZJ32)	XH112, This Study
WT Δ*rbmC* *bap1*$_{Δβ\text{-}prism\mathbf{B}+57aa}$	Δ*rbmC, bap1*$_{Δβ\text{-}prism\mathbf{B}+57aa}$*,* Δ*VC1807*::*P*$_{tac}$-*mNeonGreen*, Kan^R	Converting rugose strain to WT background by cotransformation of WT *vpvc* locus with Δ*VC1807*::*P*$_{tac}$-*mNeonGreen,* Kan^R into a rugose strain lacking *β-prism**B**+57aa* (ZJ87)	XH113, This Study
WT Δ*rbmC* *bap1*$_{Δβ\text{-}prism\mathbf{B}}$	Δ*rbmC, bap1*$_{Δβ\text{-}prism\mathbf{B}}$*,* Δ*VC1807*::*P*$_{tac}$-*mNeonGreen*, Kan^R	Converting rugose strain to WT background by cotransformation of WT *vpvc* locus with Δ*VC1807*::*P*$_{tac}$-*mNeonGreen,* Kan^R into a rugose strain lacking *β-prism**B*** (XH58)	XH114, This Study

WT Δ*rbmC* *bap1*$_{\Delta Velcro}$	Δ*rbmC, bap1*$_{\Delta Velcro}$, Δ*VC1807*::*P*$_{tac}$-*mNeonGreen*, Kan^R	Converting rugose strain to WT background by cotransformation of WT *vpvc* locus with Δ*VC1807*::*P*$_{tac}$-*mNeonGreen,* Kan^R into a rugose strain lacking *Velcro* (XH31)	XH115, This Study
**Strains Used for *in situ* Staining and Western Blots**			
bap1-3XFLAG	*vpvC*^{W240R}, *bap1-3XFLAG,* Δ*VC1807*::*P*$_{tac}$-*mNeonGreen,* Spec^R	3XFLAG tagged Bap1	JY488[9]
rbmC-3XFLAG	*vpvC*^{W240R}, *rbmC-3XFLAG,* Δ*VC1807*::*P*$_{tac}$-*mNeonGreen,* Spec^R	3XFLAG tagged RbmC	JY489[9]
Δ*rbmC bap1-3XFLAG*	*vpvC*^{W240R}, Δ*rbmC, bap1-3XFLAG,* Δ*VC1807*::*P*$_{tac}$-*mNeonGreen,* Spec^R	3XFLAG tagged Bap1 with *rbmC* deleted	ZJ065, This Study
Δ*bap1 rbmC-3XFLAG*	*vpvC*^{W240R}, Δ*bap1, rbmC-3XFLAG,* Δ*VC1807*::*P*$_{tac}$-*mNeonGreen,* Spec^R	3XFLAG tagged RbmC with *bap1* deleted	ZJ066, This Study
bap1$_{\Delta\beta\text{-}prism\mathbf{B}}$-*3XFLAG*	*vpvC*^{W240R}, *bap1*$_{\Delta\beta\text{-}prism\mathbf{B}}$-*3XFLAG,* Δ*VC1807*::*P*$_{tac}$-*mNeonGreen,* Spec^R	Replacement of *bap1*^{WT} with a *bap1* construct lacking the β-prism and containing a C-terminal 3XFLAG tag by cotransformation with Δ*VC1807*::*P*$_{tac}$-*mNeonGreen,* Spec^R into a rugose strain (JY028)	ZJ091, This Study
bap1$_{\Delta 57aa}$-*3XFLAG*	*vpvC*^{W240R}, *bap1*$_{\Delta 57aa}$-*3XFLAG,* Δ*VC1807*::*P*$_{tac}$-*mNeonGreen,* Spec^R	Replacement of *bap1*^{WT} with a *bap1* construct lacking the 57aa and containing a C-terminal 3XFLAG tag by cotransformation with Δ*VC1807*::*P*$_{tac}$-*mNeonGreen,* Spec^R into a rugose strain (JY028)	ZJ036, This Study
bap1$_{\Delta\beta\text{-}prism\mathbf{B}+57aa}$-*3XFLAG*	*vpvC*^{W240R}, *bap1*$_{\Delta\beta\text{-}prism\mathbf{B}+57aa}$-*3XFLAG,* Δ*VC1807*::*P*$_{tac}$-*mNeonGreen,* Spec^R	Replacement of *bap1*^{WT} with a *bap1* construct lacking the prism but with remaining 57aa and containing a C-terminal 3XFLAG tag by cotransformation with Δ*VC1807*::*P*$_{tac}$-*mNeonGreen,* Spec^R into a rugose strain (JY028)	XH024, This Study

bap1$_{\Delta Velcro}$-*3XFLAG*	*vpvC*^{W240R}, *bap1*$_{\Delta Velcro}$-*3XFLAG, ΔVC1807::P*$_{tac}$-*mNeonGreen,* Spec^{R}	Replacement of *bap1*^{WT} with a *bap1* construct lacking the Velcro and containing a C-terminal 3XFLAG tag by cotransformation with *ΔVC1807::P*$_{tac}$-*mNeonGreen,* Spec^{R} into a rugose strain (JY028)	XH041, This Study
bap1$_{\Delta\beta\text{-}propeller\mathbf{B}}$-*3XFLAG*	*vpvC*^{W240R}, *bap1*$_{\Delta\beta\text{-}propeller\mathbf{B}}$-*3XFLAG, ΔVC1807::P*$_{tac}$-*mNeonGreen,* Spec^{R}	Replacement of *bap1*^{WT} with a *bap1* construct lacking the β-propeller domain and containing a C-terminal 3XFLAG tag by cotransformation with *ΔVC1807::P*$_{tac}$-*mNeonGreen,* Spec^{R} into a rugose strain (JY028)	XH003, This Study
ΔhapA ΔivaP bap1$_{\Delta Velcro}$-*3XFLAG*	*vpvC*^{W240R}, *ΔhapA, ΔivaP, bap1*$_{\Delta Velcro}$-*3XFLAG, ΔVC1807::P*$_{tac}$-*mNeonGreen,* Spec^{R}	Replacement of *bap1*^{WT} with a *bap1* construct lacking the Velcro and containing a C-terminal 3XFLAG tag by cotransformation with *ΔVC1807::P*$_{tac}$-*mNeonGreen,* Spec^{R} into a rugose strain lacking *hapA* and *ivaP* (JY231)	XH057, This Study
ΔvpsL bap1-3XFLAG	*vpvC*^{W240R}, *ΔvpsL, bap1-3XFLAG, ΔVC1807::P*$_{tac}$-*mNeonGreen,* Spec^{R}	*vpsL* was deleted in the indicated strain for Western Blots	ZJ063, This Study
ΔvpsL bap1$_{\Delta\beta\text{-}propeller}$-*3XFLAG*	*vpvC*^{W240R}, *ΔvpsL, bap1*$_{\Delta\beta\text{-}propeller}$-*3XFLAG, ΔVC1807::P*$_{tac}$-*mRuby,* Kan^{R}	*vpsL* was deleted in the indicated strain for Western Blots	XH010, This Study
ΔvpsL bap1$_{\Delta 57aa}$-*3XFLAG*	*vpvC*^{W240R}, *ΔvpsL, bap1*$_{\Delta 57aa}$-*3XFLAG, ΔVC1807::P*$_{tac}$-*mRuby,* Kan^{R}	*vpsL* was deleted in the indicated strain for Western Blots	XH014, This Study
ΔvpsL bap1$_{\Delta\beta\text{-}prism\mathbf{B}+57aa}$-*3XFLAG*	*vpvC*^{W240R}, *ΔvpsL, bap1*$_{\Delta\beta\text{-}prism\mathbf{B}+57aa}$-*3XFLAG, ΔVC1807::P*$_{tac}$-*mRuby,* Kan^{R}	*vpsL* was deleted in the indicated strain for Western Blots	XH027, This Study
ΔvpsL bap1$_{\Delta\beta\text{-}prism\mathbf{B}}$-*3XFLAG*	*vpvC*^{W240R}, *ΔvpsL, bap1*$_{\Delta\beta\text{-}prism\mathbf{B}}$-*3XFLAG, ΔVC1807::P*$_{tac}$-*mRuby,* Kan^{R}	*vpsL* was deleted in the indicated strain for Western Blots	XH052, This Study
ΔvpsL bap1$_{\Delta Velcro}$-*3XFLAG*	*vpvC*^{W240R}, *ΔvpsL, bap1*$_{\Delta Velcro}$-*3XFLAG, ΔVC1807::P*$_{tac}$-*mRuby,* Kan^{R}	*vpsL* was deleted in the indicated strain for Western Blots	XH046, This Study
ΔvpsL ΔhapA ΔivaP bap1$_{\Delta Velcro}$-*3XFLAG*	*vpvC*^{W240R}, *ΔhapA, ΔivaP, ΔvpsL, bap1*$_{\Delta Velcro}$-*3XFLAG, ΔVC1807::P*$_{tac}$-*mRuby,* Kan^{R}	*vpsL* was deleted in the indicated strain for Western Blots	XH061, This Study

Δ*hapA* Δ*ivaP* *bap1*$_{\Delta Velcro\Delta 57aa}$-*3XFLAG*	*vpvC*^{W240R}, Δ*hapA*, Δ*ivaP*, *bap1*$_{\Delta Velcro\Delta 57aa}$-*3XFLAG*, Δ*VC1807*::*P*$_{tac}$-*mNeonGreen*, Spec^{R}	Replacement of *bap1*^{WT} with a *bap1* construct lacking the Velcro and the 57aa and containing a C-terminal 3XFLAG tag by cotransformation with Δ*VC1807*::*P*$_{tac}$-*mNeonGreen*, Spec^{R} into a rugose strain lacking *hapA* and *ivaP* (JY231)	STN0060, This Study
Δ*bap1*+*P*$_{BAD}$-*bap1*$_{\Delta\beta\text{-}prism\mathbf{B}}$-*3XFLAG*	*pEVS*(*P*$_{BAD}$_*bap1*$_{\Delta\beta\text{-}prism\mathbf{B}}$-*3XFLAG*, Kan^{R}), *vpvC*^{W240R}, Δ*bap1*, Δ*VC1807*::*P*$_{tac}$-*mNeonGreen*, Spec^{R}	*pEVS* containing *bap1*$_{\Delta\beta\text{-}prism\mathbf{B}}$-*3XFLAG* with an arabinose inducible promoter was mated into the indicated strain for overexpression	XH081, This Study
Δ*vpsL* Δ*bap1* +*P*$_{BAD}$-*bap1*$_{\Delta\beta\text{-}prism\mathbf{B}}$-*3XFLAG*	*pEVS*(*P*$_{BAD}$_*bap1*$_{\Delta\beta\text{-}prism\mathbf{B}}$-*3XFLAG*, Kan^{R}), *vpvC*^{W240R}, Δ*vpsL*, Δ*bap1*, Δ*VC1807*::*P*$_{tac}$-*mNeonGreen*, Spec^{R}	*vpsL* was deleted in the indicated strain for Western Blots	XH082, This Study
Δ*bap1* *rbmC*$_{\Delta\beta\text{-}prism\mathbf{C1}}$-*3XFLAG*	*vpvC*^{W240R}, Δ*bap1*, *rbmC*$_{\Delta\beta\text{-}prism\mathbf{C1}}$-*3XFLAG*, Δ*VC1807*::*P*$_{tac}$-*mNeonGreen*, Spec^{R}	Replacement of *rbmC*^{WT} with a *rbmC* construct lacking the first β-prism domain and containing a C-terminal 3XFLAG tag by cotransformation with Δ*VC1807*::*P*$_{tac}$-*mNeonGreen*, Spec^{R} into a rugose strain lacking *bap1* (JY074)	STN0049, This Study
Δ*bap1* *rbmC*$_{\Delta\beta\text{-}prism\mathbf{C2}}$-*-3XFLAG*	*vpvC*^{W240R}, Δ*bap1*, *rbmC*$_{\Delta\beta\text{-}prism\mathbf{C2}}$-*3XFLAG*, Δ*VC1807*::*P*$_{tac}$-*mNeonGreen*, Spec^{R}	Replacement of *rbmC*^{WT} with a *rbmC* construct lacking the second β-prism domain and containing a C-terminal 3XFLAG tag by cotransformation with Δ*VC1807*::*P*$_{tac}$-*mNeonGreen*, Spec^{R} into a rugose strain lacking *bap1* (JY074)	STN0050, This Study
Δ*bap1* *rbmC*$_{\Delta\beta\text{-}prism\mathbf{C1C2}}$-*3XFLAG*	*vpvC*^{W240R}, Δ*bap1*, *rbmC*$_{\Delta\beta\text{-}prism\mathbf{C1C2}}$-*3XFLAG*, Δ*VC1807*::*P*$_{tac}$-*mNeonGreen*, Spec^{R}	Replacement of *rbmC*^{WT} with a *rbmC* construct lacking both β-prism domains and containing a C-terminal 3XFLAG tag by cotransformation with Δ*VC1807*::*P*$_{tac}$-*mNeonGreen*, Spec^{R} into the wildtype rugose strain (JY28)	STN0037, This Study
Δ*vpsL* *rbmC-3XFLAG*	*vpvC*^{W240R}, Δ*vpsL*, *rbmC-3XFLAG*, Δ*VC1807*::*P*$_{tac}$-*mTFP*	*vpsL* was deleted in the indicated strain for Western Blots	JY167[8]
Δ*vpsL* *rbmC*$_{\Delta\beta\text{-}prism\mathbf{C1}}$-*3XFLAG*	*vpvC*^{W240R}, Δ*vpsL*, Δ*bap1*, *rbmC*$_{\Delta\beta\text{-}prism\mathbf{C1}}$-*3XFLAG*, Δ*VC1807*::*P*$_{tac}$-*mNeonGreen*, Spec^{R}	*vpsL* was deleted in the indicated strain for Western Blots	STN0019, This Study

Δ*vpsL rbmC*$_{\Delta\beta\text{-}prismC2}$-*3XFLAG*	*vpvC*^{W240R}, Δ*vpsL*, Δ*bap1*, *rbmC*$_{\Delta\beta\text{-}prismC2}$-*3XFLAG*, Δ*VC1807*::*P*$_{tac}$-*mNeonGreen*, Spec^{R}	*vpsL* was deleted in the indicated strain for Western Blots	STN0023, This Study
Δ*vpsL rbmC*$_{\Delta\beta\text{-}prismC1C2}$-*3XFLAG*	*vpvC*^{W240R}, Δ*vpsL*, Δ*bap1*, *rbmC*$_{\Delta\beta\text{-}prismC1C2}$-*3XFLAG*, Δ*VC1807*::*P*$_{tac}$-*mNeonGreen*, Spec^{R}	*vpsL* was deleted in the indicated strain for Western Blots	STN0026, This Study
Δ*vpsL rbmC*$_{\Delta\beta\text{-}propeller}$-*3XFLAG*	*vpvC*^{W240R}, Δ*vpsL*, Δ*bap1*, *rbmC*$_{\Delta\beta\text{-}propeller}$-*3XFLAG*, Δ*VC1807*::*P*$_{tac}$-*mNeonGreen*, Spec^{R}	*vpsL* was deleted in the indicated strain for Western Blots	STN0020, This Study
**Strains used for Complementation Assays**			
Δ*rbmC* Δ*bap1* +*P*$_{BAD}$-*bap1*	*pEVS* (*P*$_{BAD}$_*bap1*, Kan^{R}), *vpvC*^{W240R}, Δ*rbmC*, Δ*bap1*, Δ*VC1807*::*P*$_{tac}$-*mNeonGreen*, Spec^{R}	*pEVS* containing *bap1* with an arabinose inducible promoter was mated into the indicated strain for complementation	JY583, This Study
Δ*rbmC bap1*$_{\Delta Velcro}$ +*P*$_{BAD}$-*bap1*	*pEVS* (*P*$_{BAD}$_*bap1*, Kan^{R}), *vpvC*^{W240R}, Δ*rbmC*, *bap1*$_{\Delta Velcro}$, Δ*VC1807*::*P*$_{tac}$-*mNeonGreen*, Spec^{R}	*pEVS* containing *bap1* with an arabinose inducible promoter was mated into the indicated strain for complementation	XH032, This Study
Δ*rbmC bap1*$_{\Delta 57aa}$ +*P*$_{BAD}$-*bap1*	*pEVS* (*P*$_{BAD}$_*bap1*, Kan^{R}), *vpvC*^{W240R}, Δ*rbmC*, *bap1*$_{\Delta 57aa}$, Δ*VC1807*::*P*$_{tac}$-*mNeonGreen*, Spec^{R}	*pEVS* containing *bap1* with an arabinose inducible promoter was mated into the indicated strain for complementation	ZJ067, This Study
Δ*rbmC bap1*$_{\Delta\beta\text{-}prismB}$ +*P*$_{BAD}$-*bap1*	*pEVS* (*P*$_{BAD}$_*bap1*, Kan^{R}), *vpvC*^{W240R}, Δ*rbmC*, *bap1*$_{\Delta\beta\text{-}prismB}$, Δ*VC1807*::*P*$_{tac}$-*mNeonGreen*, Spec^{R}	*pEVS* containing *bap1* with an arabinose inducible promoter was mated into the indicated strain for complementation	XH080, This Study

**Strains used for *E. Coli* Protein Purification (all in NEB T7-Express or T7-Shuffle)**			
Name	Construct/Vector	Description	Reference

Bap1$_{\Delta 57aa}$-GFP$_{UV}$	Bap1 (VC_1888 residues 25-414, 473-691) in pNGFP-BC (Kawate, 2006)	Near full-length Bap1 missing 57aa loop used for crystal structure - signal peptide removed on all constructs	[11]
β-propeller**B**-GFP$_{UV}$	Bap1 β-propeller (25-316, 515-691) in pNGFP-BC	Bap1 β-propeller domain with β-prism spliced out	[11]
β-prism**B**-GFP$_{UV}$	Bap1 β-prism (316-414, 473-514) in pNGFP-BC	Bap1 β-prism missing 57aa loop	[4]
β-prism**C1**-GFP$_{UV}$	RbmC first β-prism (VC_0930 residues 505-640) in pNGFP-BC	RbmC first β-prism domain	[4]
β-prism**C2**-GFP$_{UV}$	RbmC second β-prism (823-957) in pNGFP-BC	RbmC second β-prism domain	[4]
β-prism**C2**$_{D853A}$-GFP$_{UV}$	Same with D853A point mutation	RbmC second β-prism domain with inactivating mutation in the glycan-binding pocket	[4]
RbmC$_{M1M2}$-GFP$_{UV}$	RbmC (23-220) in pNGFP-BC	RbmC M1M2 tandem βγ-crystallin domains	This Study
RbmC$_{M2}$-GFP$_{UV}$	RbmC (113-220) in pNGFP-BC	RbmC M2 βγ-crystallin domain only	This Study
StcE-X409-GFP$_{UV}$	StcE (797-898) in pNGFP-BC	C-terminal βγ-crystallin domain of *E. coli* StcE	This Study
RbmC$_{\beta\text{-propeller}}$-GFP$_{UV}$	RbmC (218-504, 641-822, with VGA linker) in pNGFP-BC	RbmC β-propeller domain with first β-prism spliced out	This Study
RbmA-GFP$_{UV}$	RbmA (31-271) in pNGFP-BC	RbmA fused to GFP$_{UV}$	This Study
GFP$_{UV}$	pNGFP-BC	GFP$_{UV}$ alone	[12]
**Strains used for Other Purposes**			
ΔrbmC Δbap1 ΔrbmA ΔpomA	*ΔrbmC, Δbap1, ΔrbmA, ΔpomA, vpvC*^{W240R}	*rbmC, bap1, rbmA* and *pomA* are all deleted. Used for VPS purification.	JY286[13]
Rg	*vpvC*^{W240R}, *ΔVC1807*::*P*$_{tac}$-*mScarlett-I*, Spec^{R}	Rugose strain with mScarlett-I fluorescent protein inserted at a non-essential site in the genome (VC1807)	JN132, This Study
Caco-2 cells		Human colonic epithelial Caco-2 cells	ATCC HTB-37

Supplementary Table 2. Primers Used in this Study.

Primer Name	Primer Sequence (5' to 3')*	Description
**Mutant constructs**		
PTN0034	ATGATCTATTCTTCTTCCATTCACTC	*rbmC* 1.5 kb arms F1
PTN0035	ATCTTTAAGTGGGTAAACTGCTATTTTG	*rbmC* 1.5 kb arms R2
PTN0014	ACTCGTTTCAACGGCTTGAGC	$rbmC_{\Delta\beta\text{-}prismC1}$ R1
PTN0015	gctcaagccgttgaaacgagtACCAATGATCTGGATGTAAAAGGG	$rbmC_{\Delta\beta\text{-}prismC1}$ F2
PTN0016	CTCACCACCCACTGCAGC	$rbmC_{\Delta\beta\text{-}prismC2}$ R1
PTN0017	gctgcagtgggtggtgagTAACGACTCATCGCTTTACTGAACTG	$rbmC_{\Delta\beta\text{-}prismC2}$ F2
PTN0008	CGAGCTGTCACCGTCATAAGG	$rbmC_{\Delta\beta\text{-}propeller}$ R1 up
PTN0009	ccttatgacggtgacagctcgCAAGCCGTTGAAACGAGTGAGTTC	$rbmC_{\Delta\beta\text{-}propeller}$ F2 middle
PTN0010	ATCATTGGTACCATTTTGCGTAGC	$rbmC_{\Delta\beta\text{-}propeller}$ R2 middle
PTN0011	gctacgcaaaatggtaccaatgatGGTGAGTCTCCAATTTTCGGTTACTC	$rbmC_{\Delta\beta\text{-}propeller}$ F3 down
PTN0045	CGGTTCGTTCAGGATTTGCGATTGcgGCGATTGGTGCTTCTGCTTCG	$rbmC_{\beta\text{-}prismC1\ D549A}$ F
PTN0046	CGAAGCAGAAGCACCAATCGCcgCAATCGCAAATCCTGAACGAACCG	$rbmC_{\beta\text{-}prismC1\ D549A}$ R
PTN0047	CGGTTCGTTCAGGATTTGCGATTGcg	detect F
PTN0057	GCTGCAGTGGGTGGTGAGGACTACAAAGACCATGACGGTGATTATAAA	$rbmC_{\Delta\beta\text{-}prismC2}$-*3XFLAG* F2
PTN0001	TTCGGCTTCATTCGTTGTGGC	*rbmC* ΔM1M2 R1
PTN0002	gccacaacgaatgaagccgaaTATGACGGTGACAGCTCGGC	*rbmC* ΔM1M2 F2

PTN0003	caaggtaaagggagtcttacaaATGACG	*rbmC* ΔM1M2 detect F
PTN0004	CACTGCCTTGCCAAGACCAC	*rbmC* ΔM1M2 detect R
PTN0053	CAATGCTCAGTCGTTTGGGTATAG	Amplify VC1807 F
PTN0054	TGTGAGACACCTATCCCAATCTAAG	Amplify VC1807 R
PTN0063	GAATTCgatccggtgattgattgagc	Amplify pBAD vector F
PTN0064	TTTAGACCTCCTGCGGCCGC	Amplify pBAD vector R
PTN0066	gctcaatcaatcaccggatcgaattcTCActtgtcatcgtcatccttgtaatcg	Flag tagged construct insert for pBAD vector R
PTN0067	gcggccgcaggaggtctaaaATGAAACAGACAAAAACGTTGACCG	*bap1-3XFLAG* insert for pBAD vector F
XH-P-003	CAATAACGCTTCACTGATCATGGTTGCCAATGACTACGAT	*bap1*$_{\Delta\beta\text{-}prism\mathbf{B}+57aa}$-*3XFLAG* F
XH-P-004	ATCGTAGTCATTGGCAACCATGATCAGTGAAGCGTTATTG	*bap1*$_{\Delta\beta\text{-}prism\mathbf{B}+57aa}$-*3XFLAG* R
XH-P-026	GTGCAACCACTGTTGATGCTGCTGGTGTTGTGACTGCTGACCA ATCACA	*bap1*$_{\Delta\beta\text{-}prism\mathbf{B}}$ F
XH-P-027	TGTGATTGGTCAGCAGTCACAACACCAGCAGCATCAACAGTGG TTGCAC	*bap1*$_{\Delta\beta\text{-}prism\mathbf{B}}$ R
XH-P-008	CTCAGGATGAAAAACGCTGGTAGACTGTATATCACTGCTGCTT GACGC	*bap1*$_{\Delta Velcro}$ F
XH-P-011	GCGTCAAGCAGCAGTGATATACAG	*bap1*$_{\Delta Velcro}$ R
XH-P-034	GCTCAATCAATCACCGGATCGAATTCTCACTTCAGCGGAACGC GAATGGTCGCT	construct insert for pBAD vector R
ZJ-P-005	GGGGTCAAAAGATTCTGCGTTTACTTCGACCACAGTACGCTAT GACA	*bap1*$_{\Delta 57aa}$ F

ZJ-P-006	TGTCATAGCGTACTGTGGTCGAAGTAAACGCAGAATCTTTTGACCCC	*bap1*$_{\Delta 57aa}$ R
ZJ-P-019	CTTGTCATCGTCATCCTTGTAATCGATA	Universal sequencer for 3xFLAG
ZJ-P-026	AGCAGCATTTTGAAAACTTCCGC	2.7kb upstream of *bap1*
ZJ-P-027	ATGAAATTCACGATAACCAGAAAACCG	2.7kb downstream of *bap1*
ZJ-P-052	GTGCAACCACTGTTGATGCTTATCTAGGATTAGAGTGGAAAACTAAAACGG	*bap1*$_{\Delta\beta\text{-}prism\mathbf{B}+57aa}$ Front F
ZJ-P-053	CCGTTTTAGTTTTCCACTCTAATCCTAGATAAGCATCAACAGTGGTTGCAC	*bap1*$_{\Delta\beta\text{-}prism\mathbf{B}+57aa}$ Front R
ZJ-P-054	GTTCCTGTGACACTGTCGAAAGTGACTGCTGACCAATCACACA	*bap1*$_{\Delta\beta\text{-}prism\mathbf{B}+57aa}$ Back F
ZJ-P-055	TGTGTGATTGGTCAGCAGTCACTTTCGACAGTGTCACAGGAAC	*bap1*$_{\Delta\beta\text{-}prism\mathbf{B}+57aa}$ Back R
ZJ-P-046	CATTTAATGACTCAAAGCACCGCACAGTCTGCCGTTTATGGCTACA	*bap1*$_{\Delta\beta\text{-}propeller}$ Front F
ZJ-P-047	TGTAGCCATAAACGGCAGACTGTGCGGTGCTTTGAGTCATTAAATG	*bap1*$_{\Delta\beta\text{-}propeller}$ Front R
ZJ-P-062	CGGTGCAGTTCCTAGTTGGTAAATAAAGATACTTCTGCCAGCCGC	*bap1*$_{\Delta\beta\text{-}propeller}$ Back F
ZJ-P-063	GCGGCTGGCAGAAGTATCTTTATTTACCAACTAGGAACTGCACCG	*bap1*$_{\Delta\beta\text{-}propeller}$ Back R
ZJ-P-060	GATGCGGAAAAAGTGAGTGAGTCT	1kb upstream of *bap1*
ZJ-P-061	CGCTGCACGGCATGATTAAAAC	1kb downstream of *bap1*
PJY140	AATCAAACCGGGCTTTAAATTTCATCTCGAC	3kb upstream of *bap1*
PJY141	CATGATATGCAACATCTACTGAAAGAGGTGCA	3kb downstream of *bap1*

PJY131	GTCACCTCTGGCCTTTATAACTGG	3kb upstream of *vpsL*
PJY132	GCTTGCAGCTCGTTATTGATACCATT	3kb downstream of *vpsL*
PJY129	ATATCCCGATCCAGTGCATGCAGC	3kb upstream of *vpvc*
PJY130	CCGGCTGATGCTTTGTGTCTAACGTG	3kb downstream of *vpvc*
***E. coli* expression construct primers (all to clone into pNGFP-BC)**		
RAO.0418	GCCGCGCCATGGGAACAACGAATGAAGCCGAAGGGTG	$\text{RbmC}_{\text{M1M2}}$ F
RAO.0549	CGCGCGCTCGAGTTAGTCACCGTCATAAGGGACAAC	$\text{RbmC}_{\text{M1M2}}$ R
RAO.0558	CGCGCGCATATGCGTTCAATGCGAGTACTGGCTTCTG	RbmC_{M2} F (use with RAO.0549)
RAO.0649	CGCGCGCCATGGGGCAGGCACTTCCAGCAAAAG	$\text{StcE}_{\text{C\_term}}$ F
RAO.0652	GCGCGCCTCGAGTTATTTATATACAACCCTCATTGACC	$\text{StcE}_{\text{C\_term}}$ R
RAO.0630	CGCGCGCCATGGCAGACGGTGACAGCTCGGCACTC	$\text{RbmC}_{\beta\text{-propeller}}$ F1
RAO.0637	GCTCAAGCCGTTGAAACGGTTGGGGCTCAAAATGGTACCAATG ATC	$\text{RbmC}_{\beta\text{-propeller}}$ F2
RAO.0638	GATCATTGGTACCATTTTGAGCCCCAACCGTTTCAACGGCTTGA GC	$\text{RbmC}_{\beta\text{-propeller}}$ R1
RAO.0453	CCGCGGCTCGAGCTCACCACCCACTGCAGCGCGTAATG	$\text{RbmC}_{\beta\text{-propeller}}$ R2
RAO.0648	CGCGCGCCATGGAAGTGGATTGTGAGTTACAGCCAGTG	RbmA F1
RAO.0580	CGCGCGCTCGAGTTATTTTTTACCACTGTCATTGACTG	RbmA R1

*lowercase nucleotides indicate overlapping sequence for SOE or nucleotide change for aa mutations

Supplementary References

1. Smith, D. R. *et al.* *In situ* proteolysis of the *Vibrio cholerae* matrix protein RbmA promotes biofilm recruitment. *Proc. Natl. Acad. Sci. U.S.A.* **112**, 10491–10496 (2015).
2. Fong, J. C. *et al.* Structural dynamics of RbmA governs plasticity of *Vibrio cholerae* biofilms. *eLife* **6**, e1002210 (2017).
3. Hau, W. L. W., Trau, D. W., Sucher, N. J., Wong, M. & Zohar, Y. Surface-chemistry technology for microfluidics. *J. Micromech. Microeng.* **13**, 272–278 (2003).
4. De, S., Kaus, K., Sinclair, S., Case, B. C. & Olson, R. Structural basis of mammalian glycan targeting by *Vibrio cholerae* cytolysin and biofilm proteins. *PLOS Pathog.* **14**, e1006841 (2018).
5. Floyd, K. A. *et al.* c-di-GMP modulates type IV MSHA pilus retraction and surface attachment in *Vibrio cholerae*. *Nat. Commun.* **11**, 1549 (2020).
6. Lin, H., Yu, M., Wang, X. & Zhang, X.-H. Comparative genomic analysis reveals the evolution and environmental adaptation strategies of Vibrios. *BMC Genomics* **19**, 135 (2018).
7. Yan, J., Nadell, C. D. & Bassler, B. L. Environmental fluctuation governs selection for plasticity in biofilm production. *ISME J.* **11**, 1569–1577 (2017).
8. Yan, J., Sharo, A. G., Stone, H. A., Wingreen, N. S. & Bassler, B. L. *Vibrio cholerae* biofilm growth program and architecture revealed by single-cell live imaging. *Proc. Natl. Acad. Sci. USA* **113**, e5337-5343 (2016).
9. Nijjer, J. *et al.* Mechanical forces drive a reorientation cascade leading to biofilm self-patterning. *Nat. Commun.* **12**, 6632 (2021).
10. Zhang, Q. *et al.* Morphogenesis and cell ordering in confined bacterial biofilms. *Proc. Natl. Acad. Sci. USA* **118**, e2107107118 (2021).

11. Kaus, K. *et al.* The 1.9 Å crystal structure of the extracellular matrix protein Bap1 from *Vibrio cholerae* provides insights into bacterial biofilm adhesion. *J. Biol. Chem.* **294**, 14499–14511 (2019).

12. Kawate, T. & Gouaux, E. Fluorescence-detection size-exclusion chromatography for precrystallization screening of integral membrane proteins. *Structure* **14**, 673–681 (2006).

13. Yan, J. *et al.* Bacterial biofilm material properties enable removal and transfer by capillary peeling. *Adv. Mater.* **30**, 1804153 (2018).
