# Supplementary material for: Vibrio cholerae biofilms use modular adhesins with glycan-targeting and nonspecific surface binding domains for colonization
Source: Nat Commun. 2023 Apr 13;14:2104. doi: 10.1038/s41467-023-37660-0 (PMC10102183; doi:10.1038/s41467-023-37660-0)
Supplement: Supplementary file 3 — Reporting Summary [file 41467_2023_37660_MOESM3_ESM.pdf]

nature portfolio

Corresponding author(s): Jing Yan

Last updated by author(s): Mar 15, 2023

Reporting Summary

Nature Portfolio wishes to improve the reproducibility of the work that we publish. This form provides structure for consistency and transparency in reporting. For further information on Nature Portfolio policies, see our Editorial Policies and the Editorial Policy Checklist.

Statistics

For all statistical analyses, confirm that the following items are present in the figure legend, table legend, main text, or Methods section.

n/a	Confirmed	
☐	☒	The exact sample size (*n*) for each experimental group/condition, given as a discrete number and unit of measurement
☐	☒	A statement on whether measurements were taken from distinct samples or whether the same sample was measured repeatedly
☐	☒	The statistical test(s) used AND whether they are one- or two-sided<br>*Only common tests should be described solely by name; describe more complex techniques in the Methods section.*
☒	☐	A description of all covariates tested
☐	☒	A description of any assumptions or corrections, such as tests of normality and adjustment for multiple comparisons
☐	☒	A full description of the statistical parameters including central tendency (e.g. means) or other basic estimates (e.g. regression coefficient) AND variation (e.g. standard deviation) or associated estimates of uncertainty (e.g. confidence intervals)
☐	☒	For null hypothesis testing, the test statistic (e.g. *F*, *t*, *r*) with confidence intervals, effect sizes, degrees of freedom and *P* value noted<br>*Give P values as exact values whenever suitable.*
☒	☐	For Bayesian analysis, information on the choice of priors and Markov chain Monte Carlo settings
☒	☐	For hierarchical and complex designs, identification of the appropriate level for tests and full reporting of outcomes
☒	☐	Estimates of effect sizes (e.g. Cohen's *d*, Pearson's *r*), indicating how they were calculated

Our web collection on statistics for biologists contains articles on many of the points above.

Software and code

Policy information about availability of computer code

Data collection	Nikon NIS-Elements AR (version 5.21.02) was used for image acquisition. For fixed monolayer staining, Leica LASX (Version 4.3.0.24308) was used for image acquisition.
Data analysis	Image analysis was performed with Nikon NIS-Elements AR (version 5.21.02). For fixed monolayer staining, image analysis was performed in Fiji/ImageJ2 (Version 2.9.0). For microbead adsorption assay, analysis was performed with Matlab (version 9.12.0.1927505 (R2022a)).

For manuscripts utilizing custom algorithms or software that are central to the research but not yet described in published literature, software must be made available to editors and reviewers. We strongly encourage code deposition in a community repository (e.g. GitHub). See the Nature Portfolio guidelines for submitting code & software for further information.

Data

Policy information about availability of data

All manuscripts must include a data availability statement. This statement should provide the following information, where applicable:

- Accession codes, unique identifiers, or web links for publicly available datasets
- A description of any restrictions on data availability
- For clinical datasets or third party data, please ensure that the statement adheres to our policy

All bacterial strains constructed as part of this work will be provided to the community upon request in a timely fashion and shipped in accordance with biosafety standards and regulations. All raw experimental data that support the findings of this study are available in the source data file.

Human research participants

Policy information about studies involving human research participants and Sex and Gender in Research.

Reporting on sex and gender	N/A
Population characteristics	N/A
Recruitment	N/A
Ethics oversight	N/A

Note that full information on the approval of the study protocol must also be provided in the manuscript.

Field-specific reporting

Please select the one below that is the best fit for your research. If you are not sure, read the appropriate sections before making your selection.

☒ Life sciences ☐ Behavioural & social sciences ☐ Ecological, evolutionary & environmental sciences

For a reference copy of the document with all sections, see nature.com/documents/nr-reporting-summary-flat.pdf

Life sciences study design

All studies must disclose on these points even when the disclosure is negative.

Sample size	No sample-size calculations were performed; sample size is determined according to the common standard in microbiological research. For example, see Nature communications, 8, 327 (2017). Data were taken from at least three independent biofilm measurements.
Data exclusions	No data were excluded from the analyses.
Replication	Data was generated from repeated experiments. Each experiment was successful and exhibited the same reproducible behavior. For example, microscopy images shown are representative of results from at least three independent results performed on different days.
Randomization	Not relevant. This study does not involve analysis that needs randomization.
Blinding	Blinding was not relevant in this study. Identical computational analysis tools were applied to all samples and were agnostic to the exact experimental conditions.

Reporting for specific materials, systems and methods

We require information from authors about some types of materials, experimental systems and methods used in many studies. Here, indicate whether each material, system or method listed is relevant to your study. If you are not sure if a list item applies to your research, read the appropriate section before selecting a response.

Materials & experimental systems

n/a	Involved in the study	
☐	☒	Antibodies
☐	☒	Eukaryotic cell lines
☒	☐	Palaeontology and archaeology
☐	☒	Animals and other organisms
☒	☐	Clinical data
☒	☐	Dual use research of concern

Methods

n/a	Involved in the study	
☒	☐	ChIP-seq
☒	☐	Flow cytometry
☒	☐	MRI-based neuroimaging

Antibodies

Antibodies used	Monoclonal ANTI-FLAG® M2-Cy3™ antibody produced in mouse, Sigma-Aldrich, A9594, Lot# SLCD6834<br>Direct-Blot™ HRP anti-DYKDDDDK Tag Antibody, BioLegend, Cat# 637311, Lot# B349457.

	Monoclonal CD44v6 antibody produced in rat, Thermo Fisher, Cat#BMS145, Lot#27238-000<br>Monoclonal Villin antibody produced in mouse, Clone 12, BD Bioscience, Cat#610358<br>Polyclonal Muc2 antibody produced in rabbit, Abcam, Cat#ab272692, Lot#GR3374627-11<br>Rhodamine Red X Donkey anti Rat, Jackson Immunoresearch, Cat#712-295-153, Lot#152319<br>Alexa Fluor 488 Donkey anti Rabbit, Jackson Immunoresearch, Cat#711-545-152, Lot#144917<br>Alexa 647 Donkey anti Rabbit, Jackson Immunoresearch, Cat#711-605-152, Lot#144451
Validation	Only commercial antibodies were used in this study. Antibody validation information can be found on manufacturers' websites.<br>ANTI-FLAG M2-Cy3: https://www.sigmaaldrich.com/US/en/product/sigma/a9594<br>anti-DYKDDDDK Tag: https://www.biolegend.com/en-us/products/direct-blot-hrp-anti-dykddddk-tag-antibody-12775<br>CD44v6: https://www.thermofisher.com/antibody/product/CD44var-v6-Antibody-clone-9A4-Monoclonal/BMS145<br>Villin: https://www.bdbiosciences.com/en-us/products/reagents/microscopy-imaging-reagents/immunofluorescence-reagents/purified-mouse-anti-human-villin.610358<br>Muc2: https://www.abcam.com/products/primary-antibodies/muc2-antibody-epr23479-47-ab272692.html<br>Rhodamine Red X: https://www.jacksonimmuno.com/catalog/products/712-295-153<br>Alexa Fluor 488: https://www.jacksonimmuno.com/catalog/products/711-545-152<br>Alexa 647: https://www.jacksonimmuno.com/catalog/products/711-605-152

Eukaryotic cell lines

Policy information about cell lines and Sex and Gender in Research

Cell line source(s)	Caco-2 cells were obtained from ATCC (a gift from Stavroula Hatzios, Yale).
Authentication	Cells were authenticated by ATCC based on morphology, doubling time, and STR profiling.
Mycoplasma contamination	Cells tested negative for mycoplasma contamination.
Commonly misidentified lines<br>(See ICLAC register)	No commonly misidentified cell line was used in the study.

Animals and other research organisms

Policy information about studies involving animals; ARRIVE guidelines recommended for reporting animal research, and Sex and Gender in Research

Laboratory animals	Wild-type CDl outbred mice of both sexes aged 4-12 weeks were used to generate enteroid monolayers. CDl IGS mice were purchased from Charles River Laboratories, Strain 022 and were bred for up to two generations within the Yale Animal Resource Center. Mice were maintained in ventilated Techniplast limit racks with ambient temperature of 22˚C and 50%±10% humidity in a barrier facility with 12 hour light/dark cycles. They were given ad libitum access to food and water.
Wild animals	No wild animals were used in this study.
Reporting on sex	Wild-type CDl outbred mice of both sexes were used in this study.
Field-collected samples	No field-collected samples were used in this study.
Ethics oversight	All procedures involving animal subjects were performed under the approval of the Institutional Animal Care and Use Committee (IACUC) at the Yale School of Medicine.

Note that full information on the approval of the study protocol must also be provided in the manuscript.
